# Effects of Stimulus Complexity on the Phonemic Restoration Effect †

**DOI:** 10.3390/audiolres16020060

**Published:** 2026-04-15

**Authors:** Nirmal Srinivasan, Sadie O’Neill, Chhayakanta Patro

**Affiliations:** 1 Department of Speech-Language Pathology and Audiology, Towson University, Towson, MD 21252, USA; 2 Boys Town National Research Hospital, Boys Town, NE 68010, USA

**Keywords:** phonemic restoration effect, stimuli complexity, perceptual continuity

## Abstract

**Background/Objectives:** Phonemic restoration refers to improved speech understanding when periodic silent interruptions are replaced by a plausible masking sound, reflecting an interaction between perceptual continuity and top-down linguistic inference. This study tested whether the magnitude and rate dependence of phonemic restoration vary systematically with stimulus complexity, operationalized using speech materials that differ in response constraints and linguistic variability. **Methods:** Young adults with normal audiometric thresholds completed an interrupted-speech identification task using five corpora spanning closed-set and open-set speech corpora. Stimuli were periodically interrupted at 2 Hz and 3 Hz with a 50% duty cycle. For each corpus and rate, interruption intervals were either left silent or filled with speech-shaped noise. **Results:** Closed-set materials yielded higher intelligibility than open-set materials across conditions. Replacing silent gaps with speech-shaped noise improved intelligibility for all corpora. Importantly, the joint influence of interruption rate and gap-filler depended on the stimulus type: rate-by-filler interactions were most evident for the open-set corpora as compared to the closed-set corpora. Keyword identification varied systematically with word position for the open-set materials, indicating nonuniform vulnerability across sentence structures. **Conclusions:** These results indicate that phonemic restoration is robust but material-dependent. Stimulus complexity shapes how temporal sampling and masking plausibility combine to support perceptual repair, and open-set, high-variability materials are particularly sensitive to these interactions.

## 1. Introduction

Human speech perception is remarkably robust, allowing listeners to extract coherent linguistic messages even when the acoustic signal is heavily degraded, partially masked, or intermittently unavailable. A powerful demonstration of this robustness is the phonemic restoration effect (PRE)—the illusion in which listeners “hear” missing phonemes that have been replaced by an appropriate masker such as broadband noise or a transient interrupting sound [1]. In Warren’s classic demonstration, replacing a phoneme within a meaningful sentence with a cough led listeners to report an intact utterance rather than detecting the deletion, highlighting that speech perception involves active inference using linguistic constraints and not merely bottom-up decoding of acoustic features. Subsequent work [2,3,4] showed that restoration can depend on the availability of contextual information—even information that follows the missing segment—underscoring a strong role for higher-level linguistic structure in shaping what is “heard”. Additionally, multimodal cues—such as simultaneous visual information—can strengthen the perceptual filling-in process, suggesting that restoration benefits from the integration of complementary sensory inputs [5].

The PRE is often conceptualized as a speech-specific instance of auditory induction (illusory continuity) in which the auditory system maintains a stable percept across interruptions when the interrupter could plausibly mask the target signal [6,7]. This perspective links restoration to auditory scene analysis (ASA): the perceptual system must group temporally separated speech fragments into a coherent “speech object” and attribute interruptions to external sources rather than to discontinuities in the target stream [8]. In contemporary terms, restoration is consistent with frameworks emphasizing top-down inference in the auditory system, where expectations derived from lexical/semantic structures guide interpretation when sensory evidence is ambiguous or incomplete [9,10]. Thus, the PRE provides a useful behavioral assay of how listeners integrate partial acoustic evidence with linguistic knowledge under missing data conditions [11].

Despite extensive evidence that the PRE depends on masker plausibility and the linguistic context, comparatively fewer studies have systematically examined how speech-material complexity influences restoration magnitude—especially when complexity is manipulated using corpora that differ in lexical constraint, talker variability, and response set size [12,13,14]. Theoretically, complexity could shape the PRE through (i) the strength and usefulness of top-down constraints and (ii) the ability to form a stable speech object across interruptions. In highly constrained materials, baseline intelligibility may be high even with silent gaps, leaving little measurable room for improvement when gaps are noise-filled—a ceiling-effect limitation [12]. Conversely, open-set, high-variability materials may increase uncertainty and reliance on top-down inference, potentially amplifying observable restoration benefits—provided bottom-up cues remain sufficient to support lexical access and perceptual grouping [13,14]. This “sufficient bottom-up support” caveat is important: overly degraded signals can disrupt the acoustic scaffolding needed to anchor inference and may reduce or eliminate restoration [15,16].

Previous research has shown that phonemic restoration reflects top-down repair that is nonetheless constrained by the quality of bottom-up speech cues [17,18], that is, the measured PRE for interrupted sentences in young normal-hearing listeners using noise-band vocoder simulations of cochlear-implant processing to systematically reduce spectral resolution. Restoration benefits (noise-filled gaps vs. silent gaps) depended on spectral fidelity: robust benefits appeared primarily at higher spectral resolutions, whereas low channel counts reduced or eliminated the effect. This finding demonstrates that top-down repair requires sufficient acoustic information to maintain speech object formation and lexical/phonetic inference, and it provides a mechanistic bridge between restoration paradigms and real-world listening difficulties through spectrally degraded channels [17].

Phonemic restoration is not merely an experimental artifact of using silent gaps. Training improves the intelligibility of interrupted speech both with and without filler noise, while the restoration benefit does not simply disappear with practice—consistent with listeners learning to exploit top-down mechanisms more efficiently rather than “learning away” the PRE [18]. In addition, perceptual organization cues contribute to restoration; for example, voice continuity manipulations can affect global intelligibility but do not necessarily eliminate the restoration benefit, suggesting that multiple cues can support repair when some grouping cues are weakened [19].

Critically, for experimental design, the converging literature—spanning foundational interrupted speech research and classic PRE studies—indicates that restoration is strongly shaped by interruption rate and duty cycle. Miller and Licklider’s foundational work [20] on interrupted speech established that intelligibility systematically depends on the interruption frequency and the fraction of speech preserved, providing a basis for later “gap filling” approaches. In classic PRE experiments explicitly using periodic gaps [15], filling gaps with noise can increase intelligibility when acoustic conditions support induction and when sufficient linguistic context remains when on/off patterns of approximately 175/175 ms and 200/200 ms were used, corresponding to interruption rates near 2.86 Hz and 2.5 Hz—which fall squarely within the 2–3 Hz regime. Among various interruption rates (1.5, 2.2, 3, and 5 Hz), ~2.2 Hz produced especially robust restoration with intelligibility away from the floor/ceiling [21]. Together, these findings motivate using 2 Hz and 3 Hz as theoretically grounded rates likely to yield strong restoration while also permitting systematic tests of how stimulus complexity modulates the PRE.

Although the PRE is commonly observed in anechoic or near-anechoic laboratory conditions, an important boundary condition is that room acoustics can change depending on whether noise filling benefits intelligibility. Srinivasan and Zahorik [22] demonstrated that the classic PRE can be reversed in a realistically simulated reverberant room (broadband T_60_ = 1.1 s): intelligibility was higher with silent interruptions than with noise-filled interruptions, likely because reverberant energy from speech effectively fills gaps and changes the perceptual allocation and masking plausibility of added noise. This result is theoretically important for the present work because it underscores that the PRE is not a fixed property of speech and noise alone; rather, it emerges from the reliability of continuity cues and object formation processes that can shift with the listening environment. While the present experiments focus on standard laboratory conditions, these findings motivate interpreting complexity effects as changes in how listeners group fragments and attribute interruptions under a given cue regime.

The present study operationalized stimulus complexity using five corpora spanning constrained closed-set materials to more variable open-set sentence materials. Stimulus complexity is used as a descriptive term to denote corpus-level differences in response constraints, lexical uncertainty, sentence structures, and talker variability. These factors were not independently manipulated; therefore, complexity is treated as a composite characteristic of each corpus rather than as a single explanatory variable. Closed-set corpora (Callsign Acquisition Test (CAT) [23], Coordinate Response Measure (CRM) [24], and Boston University Corpus (BUC) [25]) constrain lexical alternatives and response sets, often increasing baseline intelligibility and potentially reducing measurable restoration due to ceiling effects. In contrast, open-set materials (IEEE sentences [26] and Revised PRESTO (R-PRESTO) [27]) increase lexical and syntactic variability and, in the case of PRESTO-style materials, often incorporate broader talker/sentence variability that can raise uncertainty and increase dependence on top-down repair mechanisms. From a top-down inference perspective, open-set corpora may (i) increase the need for repair because interruptions delete informative segments and (ii) yield more headroom for improvement because baseline performance is less likely to saturate.

In this experiment, young normal-hearing listeners were tested using two interruption rates (2 Hz and 3 Hz) and two gap conditions (silence vs. speech-shaped noise). The PRE was quantified as the intelligibility benefit of noise-filled interruptions relative to silent interruptions. This design leverages the low-Hz temporal regime shown to support robust restoration and extends prior work by systematically examining whether restoration depends on corpus complexity under matched interruption parameters. It is hypothesized that the PRE will be smaller or absent for closed-set corpora (CAT, CRM, and BUC) relative to open-set corpora (IEEE and R-PRESTO), primarily because closed-set materials will produce higher baseline scores (silent gaps) and thus limited headroom for improvement with noise-filled gaps, and that the PRE will be largest for R-PRESTO and IEEE, reflecting increased lexical/talker variability and greater reliance on top-down repair when bottom-up evidence is interrupted. Also, the PRE will be robust at both rates but may be stronger at 2 Hz than 3 Hz if longer glimpses (and longer gaps) better support continuity inference and repair in meaningful sentences within the empirically supported low-Hz regime; alternatively, if 3 Hz provides more frequent glimpses that improve baseline intelligibility, it may reduce the measured PRE by compressing the noise–silence difference (i.e., a smaller benefit because silent-gap performance improves).

Importantly, the goal of the present study is not to demonstrate the existence of phonemic restoration or to compare overall intelligibility across speech corpora, as these effects are well established. Rather, this study asks whether the interaction between temporal interruption parameters and gap-filling conditions depends on stimulus complexity and whether such interactions differ across corpora even when the interruption rate and duty cycle are held constant. By examining rate-by-filler interactions across both closed-set and open-set materials, the present work tests the hypothesis that the temporal conditions that maximize restoration are not universal but instead depend on how speech information is distributed and integrated within different stimulus classes. This interaction-based approach allows us to dissociate baseline corpus effects from corpus-dependent modulation of phonemic restoration dynamics.

## 2. Methods

### 2.1. Participants

Twenty five young adults (mean age = 22.6 years; range = 19–30 years) participated in this study. All listeners demonstrated normal hearing, which was defined as pure-tone thresholds ≤ 15 dB HL at octave frequencies from 250 to 8000 Hz, with no interaural asymmetry greater than 10 dB HL at any tested frequency. The study protocol and all testing procedures were approved by the Institutional Review Board of Towson University. Participants received financial compensation for their involvement in this study.

### 2.2. Stimuli

Five speech corpora representing a range of linguistic and acoustic complexity were used in the experiment: the Callsign Acquisition Test (CAT), the Coordinate Response Measure (CRM), the Boston University Corpus (BUC), IEEE sentences, and the Revised PRESTO (R-PRESTO) corpus. These materials were selected to operationalize graded complexity manipulation spanning closed-set to open-set speech.

### 2.3. Closed-Set Materials

**Callsign Acquisition Test (CAT):** Individual test items of the CAT corpus are of the form “CALLSIGN NUMBER”. The callsigns were selected from a set of 18 two-syllable words taken from the military phonetic alphabet, and the numbers were from a set of seven one-syllable digits (1–8 except seven) (e.g., ECHO FIVE).

**Coordinate Response Measure (CRM):** Individual sentences in the CRM corpus are of the form “Ready CALLSIGN go to COLOR NUMBER now”. There are eight callsigns, four colors, and eight numbers (e.g., Ready CHARLIE go to BLUE SEVEN now).

**Boston University Corpus (BUC):** Individual sentences in the BUC are of the form “Name Verb Number Adjective Noun”. There are eight words in each of the categories. During each presentation, one word from each category is picked at random (e.g., BOB BOUGHT THREE BLUE CARDS).

CAT and CRM sentences follow highly constrained syntactic templates and only allow a limited set of possible responses, minimizing lexical uncertainty. BUC materials, while more naturalistic, were used in a structured closed-set response format consistent with prior studies utilizing these recordings. These corpora were expected to yield high baseline intelligibility due to their limited lexical diversity and predictable structure.

### 2.4. Open-Set Materials

**IEEE sentences:** IEEE sentences consist of phonetically balanced, semantically neutral content, requiring listeners to repeat entire sentences without predefined response options (e.g., the BIRCH CANOE SLID on the SMOOTH PLANKS).

**R-PRESTO sentences:** R-PRESTO sentences consist of high talker- and dialect-dependent sentence materials, creating substantial lexical and indexical uncertainty. These properties promote reliance on contextual, linguistic, and top-down inferential processes, making them especially well-suited for examining phonemic restoration under challenging conditions (e.g., the SAW is BROKEN so CHOP the WOOD INSTEAD).

### 2.5. Interruption and Filler Conditions

All materials were processed to create periodically interrupted versions of each sentence. Two interruption rates (2 Hz and 3 Hz) were implemented using a 50% duty cycle, producing alternating segments of speech and interruption. Each interrupted sentence was rendered under two filler conditions: (1) silent gaps, where speech segments were replaced with intervals of silence, and (2) noise-filled gaps, where missing segments were replaced with speech-shaped noise matched to the long-term average spectrum of each corpus. The noise was presented at a level 5 dB above the RMS level of the interrupted speech segments, resulting in an effective −5 dB gap-filling SNR relative to the missing speech. This approach ensured that the interrupting sound was a plausible masker while maintaining consistent overall presentation levels across conditions. All interruptions were implemented using periodic temporal gating with a 50% duty cycle at the specified interruption rates (2 Hz and 3 Hz). To avoid spectral splatter while preserving perceptually abrupt interruptions, a 2 ms cosine-squared amplitude ramp was applied at both the onset and offset of each preserved speech segment and interruption interval. This ramp duration was short relative to the on/off segment duration and did not alter the effective interruption rate. Noise-filled interruptions replaced the silent intervals and were processed using the same temporal gating and ramping parameters as the silent-gap condition.

### 2.6. Procedure

All testing was completed in one session, and all listeners were tested with the five speech corpora. During the testing session, the listeners were presented with 20 unique presentations in all possible combinations resulting in 20 (5 (speech corpus) x2 (interruption rates) x 2 (interrupting signals) different combinations of signal presentations, and the trial order was randomized for all participants. The listeners were seated individually in a sound-treated booth located in the Speech-Language Pathology and Audiology Department at Towson University. Auditory stimuli were presented diotically through circumaural headphones (Sennheiser HD 650; Sennheiser, Hanover, Germany). All stimulus files were generated in MATLAB (R2020a, MathWorks Inc., Natick, MA, USA) and routed through a Lynx Hilo professional-grade sound (Lynx Studio Technology, Costa Mesa, CA, USA) card to ensure accurate digital-to-analog conversion. All stimuli were presented at a 20 dB sensation level (SL) relative to each listener’s four-frequency pure-tone average (PTA: 0.5, 1, 2, and 4 kHz). For the closed-set speech materials, the participants recorded their responses using a touchscreen monitor positioned directly in front of them inside the booth (CAT: callsign and number; CRM: callsign, color, and number; BUC: 5 words, one from each category). For the open-set speech materials, the participants repeated the sentences verbally, and the experimenter marked whether the keywords were correct or incorrect. Each participant completed a total of 20 trials per corpus, corresponding to all combinations of the corpus (5), interruption rate (2 Hz or 3 Hz), and interrupting signal (silent or noise-filled). No feedback was provided for all the types of speech corpora. Data collection was self-paced, and listeners were encouraged to take breaks as needed to minimize fatigue and maintain consistent performance throughout the session.

### 2.7. Data Analysis

Analyses were performed with SPSS 28.0 (IBM Corp., Armonk, NY, USA). Repeated measures ANOVAs were used to investigate the effect of interruption rate (2 Hz or 3 Hz) and filler type (silence or speech-shaped noise) on speech intelligibility (percentage of keywords correctly identified) and phonemic restoration benefit (difference in intelligibility between noise-filled and silent-gap conditions). Also, speech intelligibility as a function of the position of the keyword in the presented stimuli was analyzed for the open-set materials. In addition to significance testing, effect sizes were quantified using omega squared (ω^2^), which estimates the proportion of variance in the dependent variable accounted for by each factor after adjusting for degrees of freedom. ω^2^ was selected due to its reduced positive bias in repeated measures designs. Effect sizes were interpreted using conventional benchmarks: values less than 0.01 were considered small, while values around 0.06 were considered medium, and values greater than 0.14 were considered large.

## 3. Results

**Figure 1:**
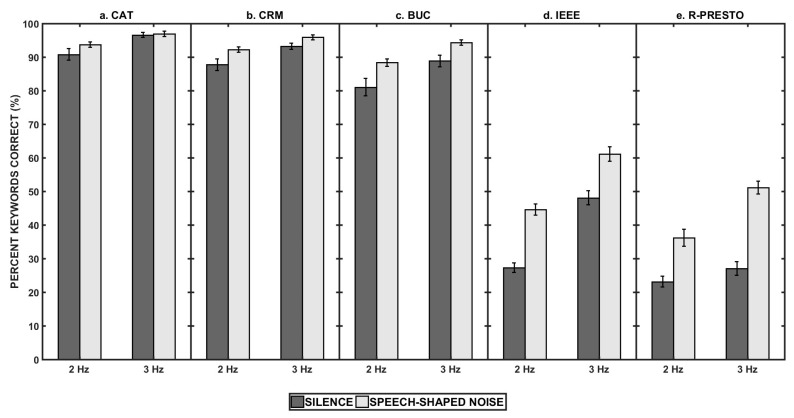
Panels (**a**–**e**) show the speech intelligibility (measured as the percentage of keywords correctly identified) for the five different corpora used in this experiment. The *x*-axis in all the panels indicates the two interruption rates used in the study, and the error bars in all the panels indicate ±1 SEM.

Figure 1 (panels (a–e)) shows the speech intelligibility for the five speech corpora used in this study. A three-way repeated measures factorial ANOVA (RM-ANOVA) was performed, with the type of response (open-set and closed-set), the interruption rate (2 Hz and 3 Hz) and the interrupting signal (silent and speech-shaped noise) as the factors and speech intelligibility as the dependent variable for the five speech corpora. There was a significant main effect of the type of response (*F*(1, 24) = 2627.20, *p* < 0.001, and ω^2^ = 0.95, indicating a very large effect) on speech intelligibility, with higher keyword identification for the closed-set corpora compared to the open-set speech corpora. There was a significant main effect of interruption rate (*F*(1, 24) = 196.65, *p* < 0.001, and ω^2^ = 0.40, indicating a large effect), and interrupting signal (*F*(1, 24) = 367.61, *p* < 0.001, and ω^2^ = 0.54, indicating a large effect) on speech intelligibility. Also, the two-way interactions between the type of response and the interruption rate (*F*(1, 24) = 34.81, *p* < 0.001, and ω^2^ = 0.21, indicating a large effect) and between the type of response and the interrupting signal (*F*(1, 24) = 132.414, *p* < 0.001, and ω^2^ = 0.31, indicating a large effect) were significant. The three-way interaction between the type of response, the interruption rate, and the interrupting signal on speech intelligibility was also significant (*F*(1, 24) = 5.94, *p* = 0.02, and ω^2^ = 0.20, indicating a large effect).

To better understand the significant interactions, separate RM-ANOVAs were performed, with the interruption rate (2 Hz and 3 Hz) and the interrupting signal (silent and speech-shaped noise) as the factors and speech intelligibility as the dependent variable for the five speech corpora. There was a significant main effect of the type of interrupting signal on speech intelligibility for all the corpora (CAT: *F*(1, 24) = 5.33, *p* = 0.03, and ω^2^ = 0.06, indicating a medium effect; CRM: *F*(1, 24) = 80.48, *p* < 0.001, and ω^2^ = 0.13, indicating a medium effect; BUC: *F*(1, 24) = 26.18, *p* < 0.001, and ω^2^ = 0.18, indicating a large effect; IEEE: *F*(1, 24) = 239.57, *p* < 0.001, and ω^2^ = 0.51, indicating a large effect; and R-PRESTO: *F*(1, 24) = 126.63, *p* < 0.001, and ω^2^ = 0.58, indicating a large effect). There was a significant main effect of interruption rate on speech intelligibility for all the corpora (CAT: *F*(1, 24) = 40.22, *p* < 0.001, and ω^2^ = 0.33, indicating a large effect; CRM: *F*(1, 24) = 84.16, *p* < 0.001, and ω^2^ = 0.10, indicating a medium effect; BUC: *F*(1, 24) = 28.11, *p* < 0.001, and ω^2^ = 0.19, indicating a large effect; IEEE: *F*(1, 24) = 144.41, *p* < 0.001, and ω^2^ = 0.49, indicating a large effect; and R-PRESTO: *F*(1, 24) = 29.50, *p* < 0.001, and ω^2^ = 0.24, indicating a large effect). Also, the interaction between interruption rate and interrupting signal on speech intelligibility was not the same for all the corpora—the interaction was significant for the open-set sentences (IEEE: *F*(1, 24) = 7.51, *p* = 0.02, and ω^2^ = 0.01, indicating a small effect; R-PRESTO: *F*(1, 24) = 13.60, *p* = 0.001, and ω^2^ = 0.013, indicating a small effect) and not significant for the CRM, BUC, and IEEE corpora (CAT: *F*(1, 24) = 3.31, *p* = 0.06, and ω^2^ < 0.01; CRM: *F*(1, 24) = 0.47, *p* = 0.50, and ω^2^ < 0.01; and BUC: *F*(1, 24) = 1.00, *p* = 0.33, and ω^2^ < 0.01). To further examine significant interactions, simple-effect analyses were conducted by comparing the restoration benefit (noise-filled minus silent conditions) across interruption rates within each corpus. These analyses revealed corpus-dependent differences in rate sensitivity for the open-set materials, with opposing rate dependencies observed for IEEE and R-PRESTO sentences. Simple-effect analysis revealed that the restoration benefit was higher at the 3 HZ rate compared to the 2 Hz rate for the R-PRESTO corpus, whereas the restoration benefit was higher at the 2 HZ rate compared to the 3 Hz rate for the IEEE corpus.

**Figure 2:**
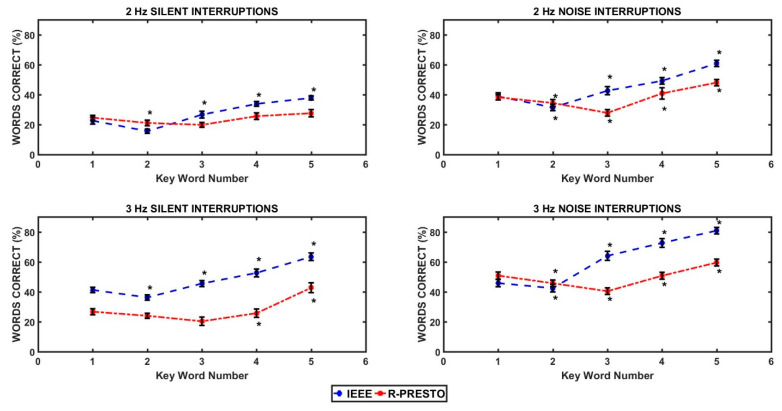
Percentage of words correctly identified as a function of word position for the IEEE and R-PRESTO speech corpora. The error bars in all the panels indicate ±1 SEM. * indicates a significant difference in words correctly identified at that position compared to the previous position.

Figure 2 shows the percentage of keywords correctly identified as a function of word position for the IEEE and PRESTO speech corpora. To understand the effect of a keyword’s position in the presented stimuli, separate one-way RM-ANOVAs with repeated contrasts were conducted to see the effect of word position on the number of keywords correctly identified for the two interruption rates and the interrupting signals. There was a significant main effect of word position on the number of keywords correctly identified for the IEEE and PRESTO speech corpora for all conditions (*p*s < 0.001). For the IEEE corpus, a U-shaped pattern was observed for all four conditions, and the word with the lowest percentage correct was word number 2. As the word position increased, the percentage of keywords correctly identified kept increasing, and the highest percentage of correct keywords was for word position 5. For the PRESTO speech corpus, the relationship between word position and percent correct was not as straightforward as it was for the IEEE corpus. For the 2 Hz silent interruptions, repeated contrasts showed no significant effect of word position on the percentage of keywords correctly identified. For the 3 Hz silent interruptions, there was no significant difference in the percentage of keywords correctly identified for word positions 1, 2, and 3. Words in position 4 had a higher identification score than words in position 3, and words in position 5 had a higher identification score than words in position 4. The relationship between the word position and the percentage of keywords correctly identified was similar for noisy interruptions at both 2 Hz and 3 Hz interruption rates, with the 2 Hz rate having a U-shaped pattern with words in position 3 and the 3 Hz rate having the least percentage of correctly identified keywords. Overall, the keywords identified as a function of word positions were ordered as follows: 1 > 2 > 3 < 4 <5.

## 4. Discussion

Although differences in intelligibility across speech corpora are well known, the present results demonstrate that corpus characteristics interact with interruption parameters in shaping phonemic restoration, rather than merely scaling overall performance. Critically, the key finding is not a main effect of corpus, but a corpus-dependent modulation of the interaction between the interruption rate and the gap-filling condition. The observation that IEEE and R-PRESTO—both open-set corpora—exhibited opposite rate dependencies for restoration indicates that the temporal conditions supporting perceptual repair vary with stimulus structure and variability. These findings extend prior work by showing that even within the same low-frequency interruption regime, the dynamics of phonemic restoration are stimulus-specific rather than universal.

### 4.1. Principal Findings

This study investigated how stimulus complexity, operationalized via five corpora spanning closed-set (CAT, CRM, and BUC) and open-set (IEEE and R-PRESTO) formats, modulates phonemic restoration under periodic interruptions at 2 Hz and 3 Hz with silent versus speech-shaped noise fillers. The results provide three clear conclusions.

First, speech intelligibility was substantially higher for closed-set than open-set materials, yielding an extremely large main effect of response type. This aligns with long-standing evidence indicating that response constraints and linguistic predictability can strongly elevate performance under degraded listening by reducing uncertainty and supporting inference [28,29]. Second, both interruption rate and interrupting signal (silence vs. noise) significantly affected intelligibility across all corpora, demonstrating that temporal sampling and interruption plausibility are foundational determinants of interrupted-speech perception [15,16,17,18,19,20,21,22]. Third—and most theoretically informative—there were significant interaction effects, including a significant three-way interaction among response type, interruption rate, and interrupting signal. Follow-up analyses localized this interaction primarily to the open-set corpora (IEEE and R-PRESTO), where the rate × filler interaction was significant and where the direction of rate dependence differed by corpus (greater restoration at 2 Hz for IEEE but greater restoration at 3 Hz for R-PRESTO). Together, these findings demonstrate that open-set materials are more sensitive to acoustic–temporal manipulations as they impose greater lexical competition and processing demands, and phonemic restoration is not a uniform “boost” produced by adding noise to gaps; rather, restoration emerges from the interplay among task constraints, speech-material properties, and temporal sampling, consistent with accounts of auditory induction and top-down inference in speech perception.

#### Stimulus Complexity and Response-Set Constraint: Why Closed-Set Outperformed Open-Set

While baseline intelligibility differences across corpora are expected given differences in response constraints and lexical uncertainty, the present findings demonstrate that these baseline differences alone do not account for the observed restoration patterns, which emerged specifically through corpus-dependent interactions between interruption rate and gap-filling conditions. Intelligibility for the closed-set corpora was consistently high across conditions, raising the possibility of ceiling effects that may have limited the sensitivity of these materials to interaction effects involving interruption rate and interrupting signal. Closed-set materials constrain the hypothesis space by restricting lexical alternatives and by providing highly stereotyped phrase structures (e.g., fixed carrier phrases and constrained response options). Under such constraints, listeners can often succeed using partial acoustic evidence (“glimpses”) without requiring full reconstruction of missing segments. This will elevate baseline intelligibility in both silent-gap and noise-filled conditions and can compress the measurable size of the PRE due to ceiling effects [30,31].

In contrast, open-set materials require lexical retrieval and sequence reconstruction without explicit response alternatives, increasing uncertainty at each word position and thereby amplifying the consequences of missing acoustic evidence. In these conditions, noise-filled interruptions can yield larger benefits because the masker plausibly explains away the absence of speech and encourages the percept of continuity, enabling higher-order inference to contribute more substantially to perception. Thus, the response-type effect likely reflects both the response constraint itself and the increased engagement of repair mechanisms under open-set demands [32,33]. These results support the methodological point that stimulus choice strongly determines whether the PRE is detectable; highly constrained corpora may underestimate restoration due to ceiling-limited improvement, whereas open-set, high-variability materials can expose interactions between temporal parameters and filler plausibility [3,32].

### 4.2. Main Effects of Interruption Rate and Interrupting Signal: Confirming Core PRE Mechanisms

Across corpora, intelligibility was significantly influenced by both interruption rate and filler type (silent vs. speech-shaped noise). This pattern is consistent with foundational work on interrupted speech showing that intelligibility systematically depends on interruption frequency and the fraction of speech preserved. It is also consistent with classic phonemic restoration demonstrations in which noise-filled gaps increase intelligibility relative to silent gaps because a plausible masker encourages the percept of continuity and supports top-down reconstruction of missing speech segments [15,16,17,18,19,20,21,22].

Importantly, the significant main effect of interrupting signal was observed in all five corpora, indicating that replacing silent gaps with speech-shaped noise yields measurable intelligibility benefits even for highly constrained stimuli (CAT/CRM/BUC), although effect sizes were substantially larger for open-set stimuli. This is consistent with the view that restoration reflects an interaction between (a) the plausibility of the interrupter as a masker and (b) the amount and utility of remaining contextual information.

### 4.3. Corpus-Dependent Rate × Filler Interactions in Open-Set Materials

The most theoretically informative outcome is that the rate × filler interaction was significant for the open-set corpora (IEEE and R-PRESTO) but not for the closed-set corpora. This suggests that temporal sampling changes the extent to which noise-filling manipulation yields a measurable restoration benefit primarily when the task requires greater lexical/semantic integration and when baseline performance is not near the ceiling.

Simple-effect analyses revealed opposite rate dependencies across the two open-set corpora: the restoration benefit was higher at 2 Hz than at 3 Hz for IEEE, but it was higher at 3 Hz than at 2 Hz for R-PRESTO. This dissociation suggests that the “optimal” interruption rate for restoration depends on how speech information is distributed in time and how rapidly contextual constraints can stabilize lexical hypotheses within each corpus.

A plausible interpretation is that IEEE sentences, which are phonetically balanced and relatively uniform in style and length, benefit from longer contiguous speech segments at 2 Hz and may better preserve the multi-phoneme and coarticulatory information needed for lexical access. Under this account, the longer glimpses at 2 Hz provide stronger anchors for reconstruction across noise-filled gaps, resulting in greater restoration benefit.

By contrast, R-PRESTO is intentionally highly variable and designed for tax speech recognition under more ecologically relevant variability. In such materials, more frequent glimpses at 3 Hz may better support incremental accumulation of evidence across talker and lexical variability, thus improving the system’s ability to maintain a speech object over time and increasing the marginal value of noise filling. Under high variability, the additional sampling opportunities at 3 Hz may therefore enhance restoration more effectively than those at 2 Hz.

This interpretation is compatible with the broader framework developed in Baskent’s work: phonemic restoration is a form of top-down repair that remains constrained by the adequacy of bottom-up information, and manipulations that reduce the effective reliability of cues (e.g., spectral degradation or high variability) can change the restoration magnitude.

The opposing rate dependencies observed for IEEE and R PRESTO materials should be interpreted cautiously. While it is plausible that differences in sentence uniformity, talker variability, and lexical structure contribute to these effects, the present design does not disentangle these factors. Accordingly, these explanations are offered as theoretically motivated interpretations rather than definitive sources.

### 4.4. Word-Position Effects: Evidence for Serial Dependencies and Contextual Build-Up

The analyses of keyword position show that intelligibility is not uniform across sentence positions and that serial position interacts with interruption parameters and corpus structure. For IEEE sentences, the observed U-shaped pattern across all conditions (lowest performance around word position 2 and increasing performance through later positions) suggests a combination of early vulnerability and later facilitation. Early words have limited preceding context to support repair, while later words benefit from accumulated contextual constraints and increased predictive structure.

R-PRESTO showed a more complex pattern, consistent with its design goal of high variability and reduced predictability. For silent interruptions, the absence of a word-position effect at 2 Hz suggests that listeners may rely more on isolated glimpses or local cues when the signal is more severely fragmented in a way that limits context accumulation. In contrast, at 3 Hz silent interruptions, later positions showed increases (positions 4 and 5 exceeding earlier positions), consistent with a gradual emergence of contextual constraints. In the noise-filled conditions, the U-shaped pattern (lowest around position 3 and increasing thereafter) suggests that the noise filler facilitates continuity and allows context-driven reconstruction to emerge more reliably across word positions. Overall, the ordering (1 > 2 > 3 < 4 < 5) supports a model where mid-sentence positions can be particularly vulnerable to interruption placement and lexical competition, while later positions benefit from contextual stabilization [34].

## 5. Limitations

Several limitations should be considered when interpreting these findings. First, the listener sample consisted exclusively of young adults with normal hearing, and the results may not be generalized to older listeners or to those with hearing impairment. Second, intelligibility for the closed-set corpora was relatively high, raising the possibility of ceiling effects that may have reduced sensitivity to condition-related interactions. Third, response and scoring formats differed across corpus types, which may have contributed to the observed differences in effect magnitude. Finally, stimulus complexity was inferred at the corpus level and reflected multiple correlated factors rather than a single experimentally isolated dimension. Future work manipulating these dimensions independently will be necessary to further specify their individual contributions to phonemic restoration.

## 6. Conclusions

This study showed that phonemic restoration is strongly shaped by speech-material complexity, response constraints, and interruption timing. Closed-set corpora yielded substantially higher intelligibility overall and smaller measurable interactions, consistent with constrained decision spaces and potential ceiling limitations. Open-set corpora revealed richer dynamics: while noise filling improved intelligibility across conditions, the interaction between the interruption rate and the filler depended on the corpus, with IEEE showing greater restoration at 2 Hz and R-PRESTO showing greater restoration at 3 Hz. These results refine classic accounts of phonemic restoration by demonstrating that optimal temporal parameters are not universal but depend on how speech materials distribute information and impose integration demands, consistent with induction-based and inference-based frameworks of robust speech perception.

## Data Availability

The data presented in this study are available on request from the corresponding author due to their inclusion in a larger dataset that is currently being analyzed.
